# TIP4.0: Industrial Internet of Things Platform for Predictive Maintenance

**DOI:** 10.3390/s21144676

**Published:** 2021-07-08

**Authors:** Carlos Resende, Duarte Folgado, João Oliveira, Bernardo Franco, Waldir Moreira, Antonio Oliveira-Jr, Armando Cavaleiro, Ricardo Carvalho

**Affiliations:** 1 Associação Fraunhofer Portugal Research, Rua Alfredo Allen 455/461, 4200-135 Porto, Portugal; duarte.folgado@fraunhofer.pt (D.F.); joao.oliveira@fraunhofer.pt (J.O.); bernardo.franco@fraunhofer.pt (B.F.); waldir.junior@fraunhofer.pt (W.M.); antonio.junior@fraunhofer.pt (A.O.-J.); 2 Laboratório de Instrumentação, Engenharia Biomédica e Física da Radiação (LIBPhys-UNL), Departamento de Física, Faculdade de Ciências e Tecnologia, FCT, Universidade Nova de Lisboa, 2829-516 Caparica, Portugal; 3 Institute of Informatics (INF), Federal University of Goiás (UFG), Goiânia 74690-900, Brazil; 4 Bresimar Automação S.A., 3800-230 Aveiro, Portugal; armando.cavaleiro@tekonelectronics.com (A.C.); ricardo.carvalho@tekonelectronics.com (R.C.)

**Keywords:** predictive maintenance, edge computing, artificial intelligence, internet of things, industry 4.0

## Abstract

Industry 4.0, allied with the growth and democratization of Artificial Intelligence (AI) and the advent of IoT, is paving the way for the complete digitization and automation of industrial processes. Maintenance is one of these processes, where the introduction of a predictive approach, as opposed to the traditional techniques, is expected to considerably improve the industry maintenance strategies with gains such as reduced downtime, improved equipment effectiveness, lower maintenance costs, increased return on assets, risk mitigation, and, ultimately, profitable growth. With predictive maintenance, dedicated sensors monitor the critical points of assets. The sensor data then feed into machine learning algorithms that can infer the asset health status and inform operators and decision-makers. With this in mind, in this paper, we present TIP4.0, a platform for predictive maintenance based on a modular software solution for edge computing gateways. TIP4.0 is built around Yocto, which makes it readily available and compliant with Commercial Off-the-Shelf (COTS) or proprietary hardware. TIP4.0 was conceived with an industry mindset with communication interfaces that allow it to serve sensor networks in the shop floor and modular software architecture that allows it to be easily adjusted to new deployment scenarios. To showcase its potential, the TIP4.0 platform was validated over COTS hardware, and we considered a public data-set for the simulation of predictive maintenance scenarios. We used a Convolution Neural Network (CNN) architecture, which provided competitive performance over the state-of-the-art approaches, while being approximately four-times and two-times faster than the uncompressed model inference on the Central Processing Unit (CPU) and Graphical Processing Unit, respectively. These results highlight the capabilities of distributed large-scale edge computing over industrial scenarios.

## 1. Introduction

Over the last few years, along with the spread of Artificial Intelligence (AI), the concepts of Industry 4.0 and Industrial IoT (IIoT) [1,2,3,4] materialised through their dissemination and implementation across several industries. With this advent, there was an increase of Predictive Maintenance (PdM) solutions based on industrial data [5,6,7,8,9]. PdM is defined as a set of techniques designed to help determine the condition of in-service equipment to estimate when maintenance should be performed through the implementation of smart scheduling preventive maintenance actions that ultimately avoid (or at least mitigate the effects of) unexpected equipment failures [10].

In a nutshell, predictive maintenance exploits the possibility of using smart machines, which can warn operators and decision-makers before they fail. The benefits include reduced downtime, improved equipment effectiveness, lower maintenance costs, increased return on assets, risk mitigation, and, most importantly, profitable growth.

According to PricewaterhouseCoopers (PwC) [11], it is estimated that PdM might reduce OPEX cost by 12%, improve uptime by 9%, reduce safety, health, environment, and quality risks by 14%, and extend the lifetime of an aging asset by 20%. Nevertheless, even in scenarios where machines are inherently producing log data regarding their normal (and abnormal) runtime, the industry has failed to adopt consolidated data analytics strategies to obtain a sense of the significant amount of data that is generated. A study conducted on manufacturing companies concluded that they experience up to 800 h of unscheduled downtime per year, 30 percent of which is unexpected. The average cost of downtime for an automotive manufacturer is $ 22.000 per minute [12].

This context supported the rise of predictive maintenance strategies, which is backed up by some recent works [5,13,14,15,16,17] in the literature proposing Internet of Things (IoT) solutions to address such challenges. In this way, edge computing is also emerging as a technology in several aspects of IIoT [18]. Topics, such as improved performance (with optimized use of communication, computation, and storage resources), data privacy, data security, reduced operational cost, real-time control, and smart decision making into legacy industrial automation and supervisory control systems are being addressed by edge computing [19,20,21].

We introduce TIP4.0, a platform for PdM based on a modular software solution for edge computing gateways. TIP4.0 is built with an industry mindset on top of a previously proposed Wireless Gateway for Industrial IoT (WGW4IIoT) [22]. The platform is flexible enough to adapt to specific installation needs and provides support for further development when new requirements emerge in industry application scenarios. While WGW4IIoT has been successfully validated in real industrial settings, it is completely agnostic to predictive maintenance, does not implement edge computing, and focuses solely in obtaining data from different sources to monitor asset operation (e.g., the temperature for industrial ceramic ovens and agro-industrial composting processes).

Furthermore, the TIP4.0 platform allows the user to personalize the models that are used for PdM so that it is possible to comply with a variety of deployment requirements and reduce the effort when transposing the platform across different shop floors and industries. Such models could also be processed on the host hardware main processor, or offloaded to a second one connected to it, such as an AI Hardware Accelerator or other unit with extra processing power.

In the scope of this work, the platform was optimized to run models over time series’ sensor data on the Google USB Edge Tensor Processing Unit (TPU) [23], providing high-speed neural network performance with a low-power cost. Prior experiments using the Edge TPU were conducted in the computer vision domain. In this work, we take Edge TPU to the next level by considering a model design to satisfactorily handle a time series. TIP4.0 allows the user to fully manage these predictive maintenance features locally even without a connection to the main network infrastructure. For such, the WGW4IIoT local web portal was improved to include the PdM feature.

In conclusion, the main contributions of this paper can be summarized as:

- A multi-purpose platform based on software and hardware components that can be selectively activated according to the deployment scenario requirements.
- A low profile software solution capable of running in hardware with reduced processing and memory power. By being based on the Yocto system, this feature allows TIP4.0 to be easily adapted to COTS or proprietary hardware.
- An industrial monitoring gateway for predictive maintenance scenarios with edge computing capabilities, local persistence storage, remote management and update features, and full autonomous operation even when disconnected from the main network.
- A modular, easily extensible software solution supporting multiple sensor and cloud protocols. Local and remote Application Programming Interface (API) interfaces are provided for the inclusion of new local and remote applications that extend TIP4.0’s functionality according to the requirements of new deployment scenarios.
- Solution developed in cooperation with an industrial monitoring service provider and product developer, making TIP4.0 aligned with the industry requirements and expectations.
- A feasibility study using the Edge TPU for running a PdM model based on neural networks with time series data.

The rest of this paper is organized as follows. Section 2 provides an overview of relevant works related to predictive maintenance and Edge Computing Gateways suitable for IIoT. This section also presents a guideline on the implementation of predictive maintenance solutions. Section 3 introduces the proposed solution, highlighting the main use cases and requirements, as well as detailing the hardware and software architectures and respective components. Section 4 presents the evaluation tests, while Section 5 concludes the paper and presents the planned future steps.

## 2. Literature Review

This section starts by presenting a relevant literature review related to predictive maintenance and a set of guidelines to realise predictive maintenance solutions. Then, we focus on the relevant literature concerning edge computing gateways suitable for IIoT.

### 2.1. Predictive Maintenance

In the past, several techniques have been used for the maintenance of deteriorating systems [24]. Such traditional techniques are now being replaced, or at least partially replaced, by predictive maintenance, due to the better results presented by this new approach [25,26], especially in Industry 4.0 [27].

PdM aims to predict failures and more accurately schedule maintenance interventions. To achieve this, sensors monitor industrial machines and communicate their data, normally, in the form of time series. By processing such data, PdM techniques aim to forecast certain rare events that represent failures. In other words, sensors monitor the tasks, or a subset of the tasks, performed by a machine. The output of these sensors represents the typical behaviour of the machine for each of these tasks, and deviations from such behaviour are identified as anomalies that might be related with the equipment degradation and corresponding failure. The identification of anomalies is then an important step for the implementation of successful PdM systems.

Carvalho et al. [28] presented a systematic review on Machine Learning (ML) methods applied to PdM. The authors concluded that ML techniques were gradually being applied for designing PdM applications with positive results. The techniques most often applied were random forest, neural networks, support vector machine, and *k*-means. In [29], a thorough overview of deep learning methods was presented in the IoT context. Despite the promising results in terms of predictive performance, the authors pointed for future research toward designing deep learning architectures for fast and real-time scenarios since these apply to many time-sensitive IoT applications.

During recent years, several architectures have been proposed in the context of PdM in a very popular benchmarking problem—the NASA Commercial Modular Aero-propulsion System Simulation (C-MAPSS) dataset. Recent architectures used Convolution Neural Networks (CNNs) [30,31], Long Short-Term Memory (LSTM) [32] or hybrid approaches [33].

One of the challenges of using deep learning approaches is their long processing time, complexity, and excessive power consumption. These issues may hinder the process of their implementation in real scenarios. Furthermore, most of the studies focused on addressing the predictive models’ performance and not discussing the model’s complexity and the inference time.

However, having a good algorithm for PdM analysis is not by itself an indication of success, due to the several aspects that must be considered by PdM solutions (e.g., the solution could be not targeting the correct assets, or it could have a low acceptance rate by the operators). Considering this, the remainder of this section compiles a set of guidelines listed in the literature that should be followed to implement predictive maintenance solutions.

#### Preparing a Predictive Maintenance Solution

When preparing a predictive maintenance solution, there is a set of best practices that should be followed to guarantee the success of the solution and the turn-over on the investment. In fact, according to Emerson, only an estimated 20% of predictive maintenance initiatives achieve the anticipated results [34]. Considering such data, we have compiled a set of best practices highlighted by Microsoft [35], Limble CMMS [36], Seebo [37], and Emerson [34] in their white-papers, blogs, and documentation for implementing predictive maintenance solutions.

Emerson [34] started by highlighting the importance of understanding that the realization of a successful predictive maintenance solution is tightly related with the recognition of the numerous elements involved in establishing a successful solution. Following a systematic process of developing, implementing, managing, measuring, and continuously improving the monitoring effort at the deployment site is the mindset that should be followed. Following this rationale, Microsoft [35], Limble CMMS [36], and Seebo [37] presented the development of a predictive maintenance solution as being composed of several steps.

Identifying the problem that should be resolved with predictive maintenance is the first step. Despite being normally disregarded, this is one of the most crucial steps in building PdM solutions, because PdM is not the best solution for all maintenance problems. For example, the cost of deploying and maintaining a PdM solution needs to compensate with the gains obtained by the reduction in the cost of equipment repair or replacement. The set of characteristics that a PdM problem should have are the following:

- The failure should be predictable, and a plan of action should be available to avoid it once it is predicted.
- Domain experts capable of understanding the information referred in the previous point should be part of the solution development. Their involvement will be important to help data analysts to understand and interpret the data, as well as to identify which additional data should be collected to better characterise the problem.

In addition to the identification of the correct problems with which PdM can be more effective, it is also crucial to have a good dataset that can be used to build and train PdM algorithms:

- Only relevant data for the problem to predict should be included in the dataset, i.e., data not directly related with the problem or not important for the prediction purpose should not be present in the dataset. To ease the creation of a good dataset for PdM, the prediction should be focused on specific components, instead of larger subsystems. As mentioned above, the domain experts should be part of the solution development to help in the definition and creation of a good dataset for PdM.
- Having a dataset with sufficient data that represent the events to predict and its causes, if possible with multiple records of such events, is also important.

The next steps are related to the algorithm creation—data preparation, feature extraction, modeling, training, validation, and testing. Once the algorithm is theoretically validated, the final step is to deploy the predictive maintenance solution in the business system and validate it (and the algorithms) with new and unseen data. An assessment of the benefits and of the reduction on the maintenance cost can then be calculated with the deployed solution.

**Figure 1:**
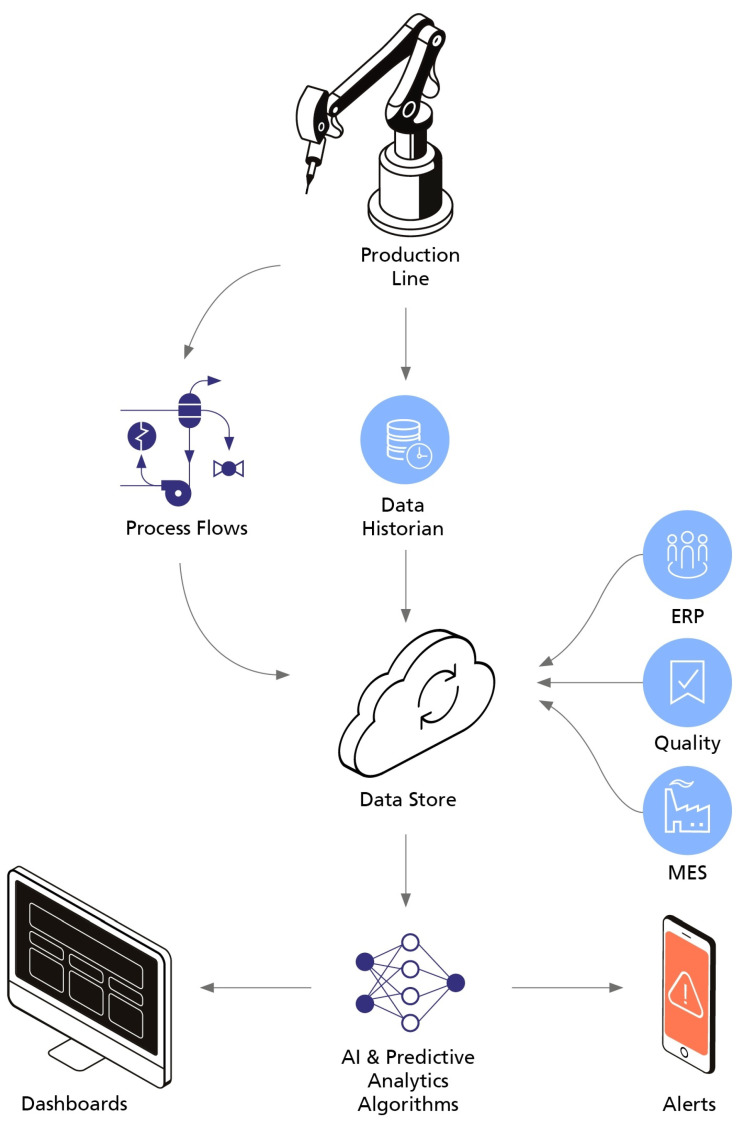
Predictive maintenance architecture (Adapted from [37]).

In the end, a solution with an architecture similar to the one presented in Figure 1 should be obtained. In this solution, machine data is streamed from the sensors to a central repository, raw or pre-processed, depending on the chosen strategy. Business data from the company Enterprise Resource Planning (ERP) and Manufacturing Execution System (MES) systems, together with other manufacturing data, are integrated into the data repository to provide context to the monitored machine data (which, again, could already be pre-processed). On top of this, predictive monitoring algorithms are applied, and the results and alerts are displayed to the user on intuitive dashboards.

### 2.2. Edge Computing Gateways Suitable for IIoT

In order to fulfil the IIoT vision and address the predictive maintenance topic, a modular gateway with AI capabilities and the robustness and communication interfaces expected in an industrial deployment is mandatory. The Bosch white-paper [38] highlighted eleven features to consider when selecting edge computing solutions:

1. Support for device protocols.
2. Local persistence storage.
3. Autonomous operation while disconnected.
4. Remote management and update.
5. Local applications.
6. OpenAPI for remote applications.
7. Analytics and machine learning.
8. Rule Engine.
9. Security and privacy.
10. Availability and reliability.
11. Device abstraction and digital twin.

There are several gateways solution proposed in the literature that attempted to address the aforementioned challenges. However, it is worth noting that our intention is not to provide an exhaustive survey on IIoT gateways. Instead, we want to discuss on relevant industrial IoT edge computing gateways solutions that are, at different extents, related to our proposed TIP4.0. For more detailed surveys on the IIoT edge computing gateways, we point the reader to [19,39,40].

Cerquitelli et al. (2019) [41] proposed a lightweight and scalable gateway architecture based on micro-services and Docker. The proposed solutions allows for the easy addition of new functionalities due to the use of a micro-service architecture. However, the reported implemented functionalities were the ones related to the basic operation of a gateway: collecting data from shop floor sensors and other systems, and relaying it to the cloud. Theoretically, other important features for industrial gateways, such as the ones identified by Bosch, could be added as services; however, these were not reported in the proposed gateway.

On the other hand, Civerchia et al. (2017) [5] described a full stack solution composed of sensors, a gateway, and a remote server, with the objective of remote monitoring of industrial machinery. Despite being validated under real conditions on a electricity power plant, the element of interest for our work, the gateway was fitted to the described application scenario. Such a lack of modularity does not allow the proposed gateway to be easily adjusted to new application scenarios and the heterogeneity of sensor and remote server communication technologies and protocols that can be found in other deployment environments.

Additionally, despite a reference for a possible inclusion of data processing units, the reported solution does not implement it. It has local storage, but no information is provided on how to access it locally, or if the gateway could be fully managed locally when disconnected from the main network, i.e., with no connection to the cloud or a remote server (through, for example, a local web server and web interface provided via a management Ethernet port, a user interface available in a monitor connected to the gateway, or a Desktop or Mobile application that directly accesses the gateway). Considering this, the feature of autonomous operation while disconnected is not supported, because, without the local management functionality, it will not be possible to correctly manage the gateway; therefore, it will not be fully functional.

A edge solution for the predictive maintenance of pumping equipment was proposed by Short and Twiddle (2019) [42]. Like the previous work, the proposed embedded platform supported multiple industrial communication interfaces but was limited to the described application scenario and did not present any features to allow it to be easily adapted to new contexts.

Yamato et al. (2016) [43] also proposed a complete maintenance platform. The proposed solution was based on edge nodes and cloud services that together were able to analyse sensing data and detect anomalies. The cloud element was responsible for the analysis of the data in detail and the update of the learning models running on edge nodes, thereby, improving its analysis accuracy. Despite presenting a very interesting solution for predictive maintenance, the solution was solely focused on the AI part of predictive maintenance, and thus no attention was given to the architecture of the gateway.

Such a focus was presented by Lojka et al. (2016) [44] and Zolotová et al. (2015) [45], where the authors proposed new concepts for an industrial gateway architecture with modularity, flexibility, efficiency, and robustness in mind. These promising proposals addressed the majority of the features highlighted in the Bosch white-paper [38]; however, in both publications, the authors presented a very embryonic solution, that, from the presented concept, implemented only the basic functionalities of the gateway.

Craciunescu et al. (2020) [46] presented an IIoT gateway for edge computing applications. However, despite their statement regarding the versatility of the solution for different industrial scenarios, no information was given on how to achieve this, neither on how to add extra functionalities to the gateway nor on how to extend the existing communication interfaces. From the application examples provided, the processing capacity of the gateway was very limited, and no information was provided about the remote management and autonomous operation if disconnected from the main network.

Alam et al. (2018) [47] presented a solution for IoT applications focused in the different layers of the IoT architecture. They proposed the combined use of Docker and micro-services. Despite being a very interesting solution, when comparing its features with the aforementioned ones as crucial for a gateway targeting edge computing on industrial application, it was possible to identify the lack of support for a considerable set of them.

On the other hand, Zhong et al. (2015) [48] proposed a solution focused on the gateway itself, which discussed a concept of layers that adds modularity to the solution. Nevertheless, the proposed solution lacked a more robust validation process, and it was missing the essential details for an edge computing gateway for industrial application, namely the remote management, embedded AI, local storage, and autonomous operation while disconnected.

Kang et al. (2017) [49] proposed a new self-configurable gateway solution that featured real time detection and the configuration of smart things; automatic updates of hardware changes; and the connection management of smart things. The proposed solution was very dynamic and allowed for a fast and autonomous adaptation to the installation context but was lacking some details about its implementation. When compared to the features of a edge computing gateway for industrial application, it was lacking the flexibility in terms of new features and applications running on it as well as remote management and an embedded AI.

Rahmani et al. (2018) [50] presented a gateway for e-Health services with features, such as local storage, real-time local data processing, and embedded data mining, thus, covering some of the features listed above. They explored the strategic position of the gateways at the edge of the network to exploit the concept of Fog Computing, forming a geo-distributed intermediary layer of intelligence between the sensor nodes and the Cloud. However, the proposed solution does not fit directly into the industrial use case, because the proposed features were not though with such requirements in mind. As an example, the local database and storage feature was focused on fog computing and not on a fully autonomous operation while disconnected from the main network.

On top of the diversity of gateway solutions available in the literature, as presented in this chapter, there are a variety of architectures proposed for industrial edge computing, both with academic and industrial origin, as pointed by Candanedo et al. (2019) [51] and Ojanpera et al. (2019) [40]. Such architectures define the different abstraction levels for a complete edge computing and Industry 4.0 system, from the sensors to the cloud. It is defined how each layer relates with the others, which elements are contained in each layer, and which role the element plays in that layer. As pointed out by the authors, the number of layers, the device and features contained in each of them, and the way each layer complements the others to build the complete edge computing and Industry 4.0 system are very different between each proposal.

Nevertheless, the definition of a common, or set of compliant architectures, is crucial for the interoperability required in the Industry 4.0 vision. According to [40], such compliance is being developed between some of the proposed architectures (mainly between government/industry led proposals: the German RAMI4.0, the Industrial Internet Consortium proposal, the Japanese Industrial Value Chain Reference Architecture, the Chinese Intelligent Manufacturing System Architecture, the South Korean Government Manufacturing 3.0 project and Smart Factory initiative, and the France and Italy Alliance Industrie du Futur and Piano Impresa 4.0) but not between all of them.

With this in mind, an industrial gateway could face a deployment scenario composed by any of the architectures studied in [40,51] or a new proprietary implementation. In this heterogeneity of possible deployment scenarios, the modular industrial gateway proposed by TIP4.0 platform comes into hand.

This brief overview allows the reader to see the relevance of IIoT edge computing gateways and, most importantly, to conclude that a solution that fully complies with the PdM and industrial edge computing requirements is yet to be conceived. Some solutions targeting industrial application scenarios are presented; however, they only partially address the PdM and industrial edge computing requirements. If compared with the Bosch white-paper [38] on edge computing, only a small subset of the listed requirements are met.

The same applies for the solutions focused on edge computing in general, with no special consideration for its application in industrial environments. In this case, there is also, as expected, a lack of focus on industrial scenarios. The solutions proposed by Lojka et al. (2016) [44] and Zolotová et al. (2015) [45] are the ones more aligned with the PdM and industrial edge computing requirements; however, the presented solutions are in an embryonic stage. These detailed the concept of the solution, but only a small subset was implemented.

Additionally, the work presented by Candanedo et al. (2019) [51] and Ojanpera et al. (2019) showed that an industrial gateway for the IIoT era needs to be highly modular and adaptable to meet the heterogeneity of possible deployment scenarios and Industry 4.0 architectures.

In the next section, we present our proposal describing how to address the aforementioned challenges focusing on a complete solution for IIoT edge computing.

## 3. Our Proposed TIP4.0

With an understanding on what predictive maintenance is, its various facets, how to implement it, and a literature review on edge computing solutions suitable for IIoT, this section presents TIP4.0.

TIP4.0 is focused on the development of a modular platform for predictive maintenance, which can be tailored to specific deployment scenarios. Relevant features that compose TIP4.0 modular capabilities are: (i) the selective activation of the correct software and hardware components demanded by the deployment scenario requirements; and (ii) the use of APIs for the creation of new local and remote application that complement the already existing functionalities.

**Figure 2:**
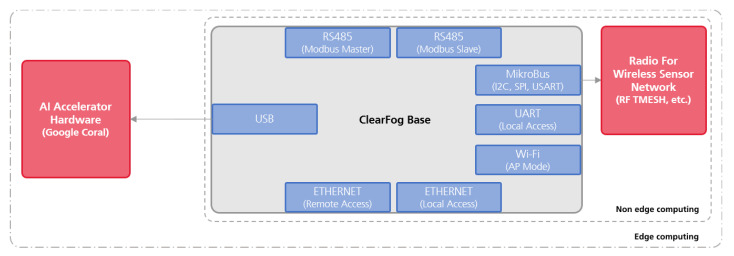
TIP4.0 hardware architecture.

This modularity is also extended to the hardware platform it supports. TIP4.0 builds on top of a customised operating system built with Yocto. Due to the Yocto support, TIP4.0 is natively modular regarding the hardware platform in which it is running. To add support for a new hardware platform, the Yocto based operating system simply needs to be compiled for the new hardware architecture. Within the scope of the presented work, we considered a ClearFog Base [52] Single Board Computer (SBC), as detailed in Figure 2; however, other hardware platforms could be added.

When compared to the solutions presented in Section 2.2 and when considering the features mentioned in the Bosch white-paper for selecting edge computing solutions, TIP4.0 proposes a more complete solution that fully supports eight of those features, namely 1–8; and partially supports other two, namely 9–10. With this approach, TIP4.0 is closer to the ideal edge computing solutions presented by Bosch, since it implements a wider number of features than its state-of-the-art counterparts. Feature 11 is left out of the current TIP4.0 implementation, because it is dependent on the Cloud platform to which TIP4.0 is connected, and, in the current deployments, the considered cloud platform does not support the digital twin feature.

Additionally, TIP4.0 also distinguishes itself due to the modularity it offers with a set of APIs to integrate new software modules, both in the edge (i.e., inside the gateway using a ZeroMQ socket or the Core API) or in the cloud (using a ZeroMQ socket or a Rest API). This allows it to be tailored to the Industry 4.0 architecture present at each deployment, and extended to support the feature set demanded by the application scenario. More details on ZeroMQ socket, Core API and Rest API are available in Section 3.2 and in [22].

In TIP4.0, we focused on the AI-related features of the ideal edge computing solution—Analytics and machine learning and Rule Engine—since the others were inherited from WGW4IIoT. Particularly, the support to the AI features was added considering a set of requirements identified by the authors according to the envisioned application scenario and Bresimar Automação S.A. strategy for their product portfolio, namely: 

Performance Requirements

- Multiple algorithms running concurrently.
- Possibility to instantiate the same algorithm multiple times.

Security Requirements

- User management and authentication layer is re-used from WGW4IIoT.
- Only system admins are allowed to make modifications to the database. All other users can only view the data.

Software Quality Attributes

- The system, software plus hardware, must be developed in such a way that it can be adapted in a modular manner for predictive maintenance with and without edge computing requirements. The objective is to reduce maintenance overhead by having a single solution based on modules/plugins that can be activated (or added in the case of hardware features) depending on the deployment scenario.
- On the fly algorithm updates, i.e., no need for operating system updates or a system reset.
- The hardware and software architecture should be ready for edge, edge+cloud, and cloud computing deployments.

With this in mind, TIP4.0—a shop floor gateway to be integrated in Industry 4.0 scenarios to achieve predictive maintenance based on AI—was designed and implemented. An overview of its hardware and software is presented next.

### 3.1. TIP4.0 Hardware Architecture

Figure 2 depicts an overview of a TIP4.0 hardware solution. This shop floor gateway is composed of the ClearFog Base [52] Single Board Computer (SBC), to which is added an AI Accelerator Hardware. Such an element can be composed by any of the AI accelerator solutions available in the market [23,53,54,55]; however, for the scope of this work, we considered the Google Edge TPU Coral [23]. Its USB and Mini PCIe interfaces, along with the native support to the ML library TensorFlow (https://www.tensorflow.org/, accessed on 30 June 2021) were some of the reasons for the selection of this AI accelerator.

The hardware support for major operative system distributions, as well as the documentation on how to add its support to Yocto, and the C++ and Python API were also relevant features considered when choosing this platform. In addition to the AI Accelerator Hardware, which is materialized in the USB version of Google Coral, TIP4.0 contains a list of interfaces, native to the ClearFog Base SBC and added with extra hardware, namely: the RS485, to access to the Modbus RTU Master and Slave instances; the Ethernet and Wi-Fi, for local/remote access to the board and Modbus TCP (TCP version of the Master and Slave Modbus instances); and MikroBus, for expanding the solution with extra wireless radio interfaces that communicate with the shop floor sensors (Figure 2 lists TMESH, the Tiny Mesh protocol provided by RadioCraft (https://radiocrafts.com/products/tinymesh/, accessed on 30 June 2021).

The two deployment scenarios addressed by TIP4.0 are also depicted in Figure 2, where the edge computing scenario is highlighted by including the hardware component used on it—the AI Accelerator Hardware—and the non-edge computing scenario removes such components from its set.

Supporting this hardware, a set of software modules was developed. The software architecture under which these software modules were developed, as well as their implementation details, are presented next.

### 3.2. TIP4.0 Software Architecture

**Figure 3:**
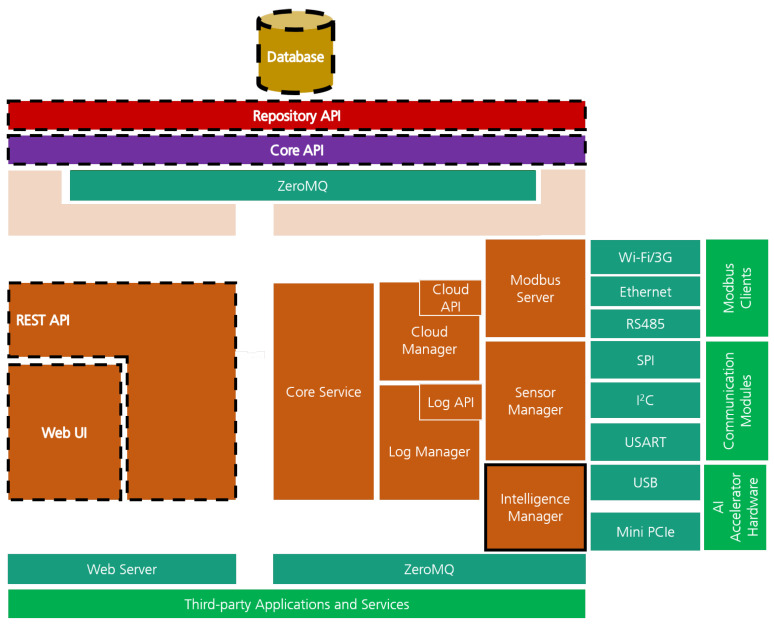
TIP4.0 High-level system architecture. Dashed black outline identifies the updated software modules, while the solid black outline identifies the new software module.

Figure 3 presents TIP4.0 software architecture, highlighting the relevant system’s elements where TIP4.0 demanded a new implementation. These modules (highlighted with a solid/dashed black outline) are: the Intelligence Manager module, a new software module responsible for interfacing with the AI accelerator hardware; the database, adapted to include the new edge computing features; the database access APIs (Repository API, Core API, and Rest APIs) updated to provide access to the new feature of the system database; and the Web UI where the new features were exposed to the user. As the remaining elements were inherited from WGW4IIoT, we point the reader to [22] for further details.

The remaining of this section details the implementation of each of these components.

#### 3.2.1. Updates to Database, Core API, Repository API, and Rest API

TIP4.0 edge computing features impose some changes to the system data model—namely, the inclusion of new entities on the system database, and the implementation of the access to those entities in the Core and Repository API (that expose the new entities to the internal software modules), and REST API (that expose the new entities to the outside, including to the web interface). The entities added to the system data model in the scope of TIP4.0 are detailed next.

Model—a new table in the database responsible for storing the information about the ML models. It stores information, such as the model name, the path where the model is stored inside the TIP4.0 shop floor gateway, and the model metadata. This metadata gives the system information about the model itself, namely: its inputs, including the type of data it expects to receive (e.g., temperature, noise, and others), the frequency of that data; and its outputs, including the type of the outputs (e.g., the number of cycles until failure) and the frequency at which the output should be generated.

This set of information is what we call the Model, and with it the system is capable of identifying if a certain sensor, its data type, and sampling frequency are compatible with a certain model, and provides an error otherwise. This also allows understanding of if the model is being correctly fed with data, or if a sensor has failed and it is not feeding the model with the correct frequency.

Another important detail is the fact that the Model output is characterized in such a way that allows the system to use it as sensor data. This approach allows not only to use the output of a Model directly as the input of another Model but also allows the cloud manager to see such data as sensor data and directly transmit them to the active cloud services, where they can also be stored or serve as inputs of another model. More details on how this feature is supported are provided on Section 3.2.2.

Predictive Analysis—a new table in the database used to associate a certain Model to different contexts, i.e., to the different deployments were PdM with edge computing is needed and can be addressed by that particular Model. For example, in two parallel production lines with two equal machines (Machine A and Machine B) submitted to PdM, the same Model will be applied to both machines, creating two different predictive analyses: the Machine A Predictive Analysis and Machine B Predictive Analysis. Predictive Analysis allows not only the association of Models to different contexts but also the aggregation of sensors in groups that compose such contexts and feed the Model.

Predictive Analysis was created in a way that allows the system to look at them as a new sensor (a synthetic sensor) and, thus, can be linked together to create more complex scenarios. For example, a set of predictive analyses could be monitoring certain parts of a machine, and a higher level predictive analysis that groups all the part-specific predictive analyses monitoring parts of the machine would be monitoring the machine itself. This same example can be extrapolated to a set of predictive analyses monitoring machines in a production line and their groups the production line itself.

Along with the introduction of the Model and Predictive Analysis in the system data model, the corresponding access API (Core API, Repository API, and Rest API) were updated to include the new data model entities. With this inclusion, the APIs expose the CRUD (Create, Read, Update, and Delete) operation of the new entities, allowing new software modules, running either in the gateway or in the cloud, to build new features on top of TIP4.0.

Additionally, both Model and Predictive Analysis were added to the system in a way that allows it to correctly function when they are not needed, i.e., in scenarios without edge computing requirements, the TIP4.0 will not use these entities; however, no special configuration is needed regarding deployment for it to work correctly.

#### 3.2.2. TIP4.0 Intelligence Manager

The Intelligence Manager module is the element responsible for feeding data to the ML models and collecting the inference results. The Intelligence Manager module uses the shell and a named pipe to pass data, thus, ensuring transparency and ease of use.

In the TIP4.0 framework, the models, as explained in Section 3.2.1, are an archive of files that include a metadata file, a shell script, and the model files. The metadata file specifies the inputs and outputs of the model and some user information. The shell script handles a set of commands that will be issued by the Intelligence Manager module to interact with the model files.

The model files include pre-processing and inference mechanisms and can be Python scripts using state-of-the-art libraries, such as: Tensorflow and the corresponding compiled models; a C program with some native inference library; or even a shell command since the Intelligence Manager module interacts with them using a shell script and a named pipe. The use of this strategy allows the Intelligence Manager module to be agnostic regarding the AI Accelerator Hardware used, since it relegates that interaction to each model. If the SBC computational power is capable of handling the model, the Intelligence Manager module can also use it to run the model without any extra hardware.

To be used by the models, the libraries (or other dependencies) need to be integrated in the SBC OS image. The current scenario fully supports TensorFlow Lite and corresponding Python libraries.

Another feature of the Intelligence Manager module is its modularity regarding the machine where it is running. The Intelligence Manager module is capable of running both inside the TIP4.0 shop floor gateway and also on the cloud. As long as the access to the ZeroMQ configuration and data sockets is guaranteed, there is no requirement for the Intelligence Manager module to be running on the same hardware as the other modules of the gateway.

**Figure 4:**
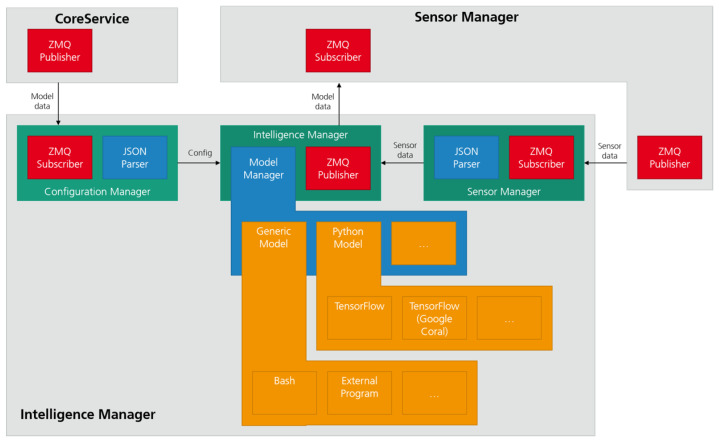
Intelligence Manager high level block diagram.

Figure 4 gives an overview on how the Intelligence Manager is organized. It uses the Configuration Manager entity to listen on the ZeroMQ (ZMQ) socket for the module configurations—namely, the models and corresponding sensor attributions. On the other end, it uses the ZMQ Sensor Manager entity socket to listen for sensor data. The Intelligence Manager entity routes this sensor data to the correct models and publishes on another ZMQ socket the results from the inference. These results are consumed by the Sensor Manager module and repackaged as sensor data. This allows the system to feed this data to models, thereby, allowing the creation of complex inference pipelines.

#### 3.2.3. Web Portal and Interface

A core functionality of TIP4.0 is its capability to work offline. Part of this feature is supported by a local web interface that allows the user to completely configure TIP4.0, as well as to visualize the monitored data. On the scope of TIP4.0, the web interface allows the user to completely manage TIP4.0 new entities (Models and Predictive Analysis) and see each Predictive Analysis result.

Regarding the Models, the user can upload, delete, and edit them. The upload needs to contain a valid Model (according to what is specified in Section 3.2.1). The Model deletion and update process needs to comply to a set of rules to guarantee the system integrity, namely:

- A Model can only be deleted if it is not being used in Predictive Analysis. If the Model is already in use, the system will warn the user about that, and ask him to first delete the Predictive Analysis in which it is being used.
- Updating a Model to a new version replaces it for all Predictive Analysis where the Model is being used. To guarantee that the update will not break the Predictive Analysis already configured, the new Model is required to use the same inputs and expose the same outputs, i.e., the metadata of the new Model needs to be exactly the same as the metadata of the old model. Otherwise, no update is performed.

For Predictive Analysis, the web interface allows the creation, update, deletion, and visualization of the the Model results. A set of rules are also enforced to guarantee system integrity, namely:

- The sensor-to-Model association is done per Model input, and, to guarantee that the Model will be correctly fed with data, the system only allows the association of sensors compatible with the selected Model input; therefore, incompatible sensors will not be displayed for selection.
- When deleting and updating the Predictive Analysis, a warning message is displayed to the user to guarantee that the update will not harm the system configuration.

**Figure 5:**
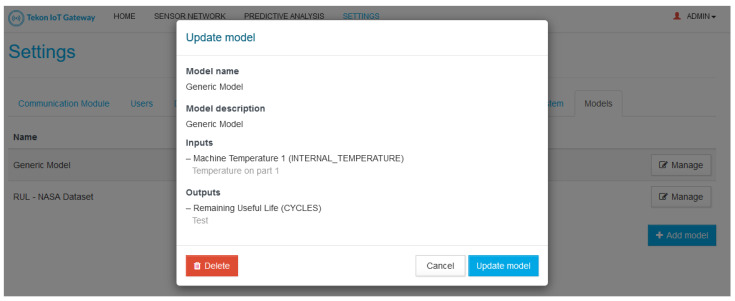
Model details.

**Figure 6:**
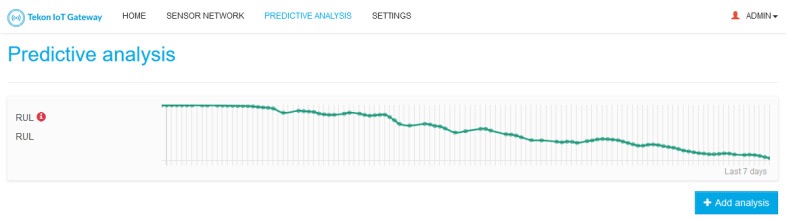
Predictive Analysis List. In this screen, the TIP4.0 web interface lists the available Predictive Analysis along with a summary of their output. In the example, only one Predictive Analysis is available, the RUL—Remaining Useful Life, and the graph depicts its evolution over time.

**Figure 7:**
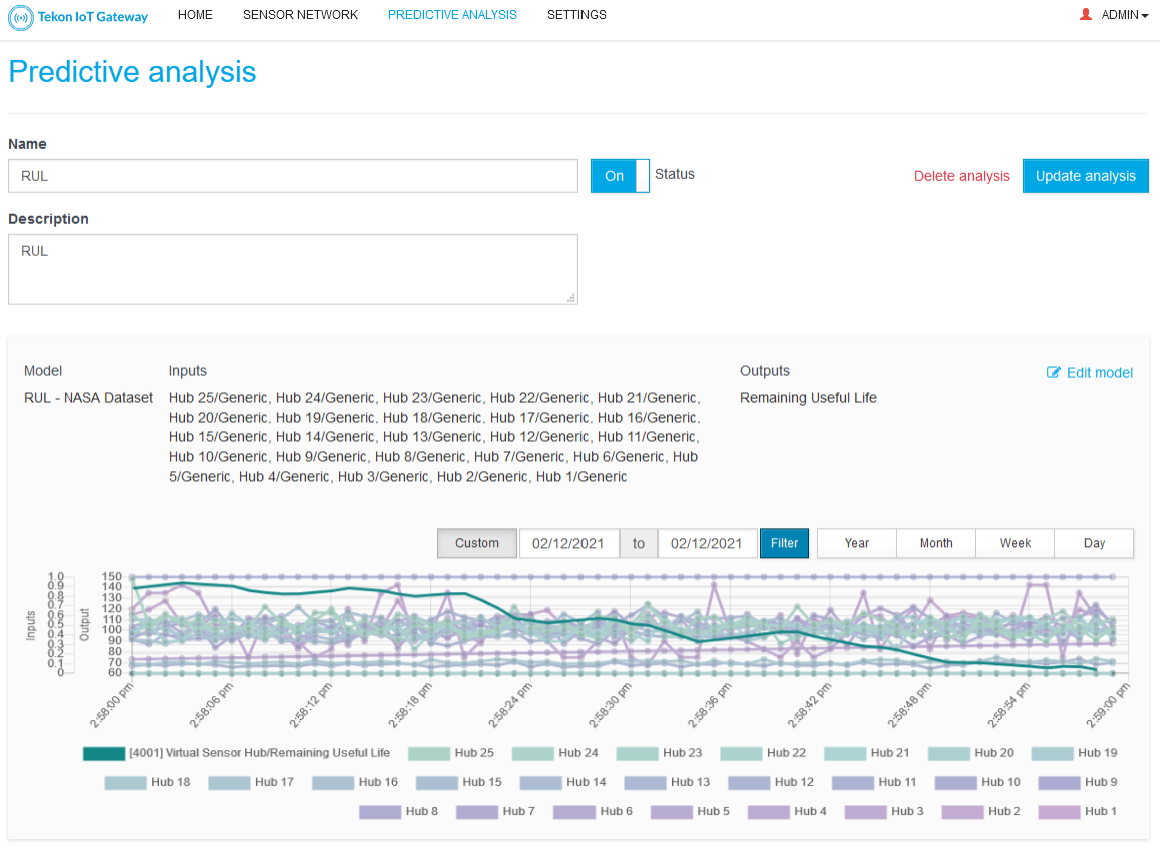
Predictive Analysis Details. In this screen, the TIP4.0 web interface lists the Predictive Analysis name, description, status, Model, and Model configuration (inputs and output data). A plot with the behaviour over time of the sensor used as input for the Predictive Analysis along with the corresponding Model output is also displayed.

Figure 5, Figure 6 and Figure 7 presents some examples of the mentioned web interface screens. The web interfaces are named Tekon IoT Gateway as this is the commercial name used by Bresimar Automação S.A. for TIP4.0 and the solution presented in [22].

## 4. Validation

The scheduling of corrective maintenance uses PdM techniques as a tool to help in the anticipation of equipment failures. With such tools, maintenance teams can improve their maintenance procedures and reduce the events of unexpected equipment downtime, along with the corresponding cost and costumer dissatisfaction that rises with them. The use of such approaches to improve maintenance procedures started in the 2000s, when the manufacturing industry—more precisely, the semiconductor manufacturing industry—started applying PdM techniques to deal with the data generated in their manufacturing settings.

PdM attempts to define the health factor or the status of a process (e.g., the remaining useful life of such a process or machine). To accomplish this, ML techniques are commonly employed, and their inference results feed into a Support Decision System that manages maintenance interventions [56].

Hence, this section summarizes the results where the main objective was to evaluate the feasibility of using the Coral USB Accelerator to process time series data, as well as, its integration in TIP4.0.

This section presents the dataset used for the model validation (Section 4.1), the preprocessing steps needed to compute the data (Section 4.2), an overview of the developed model (Section 4.3), and the obtained inference time results (Section 4.4).

### 4.1. Dataset

We used the NASA C-MAPSS dataset [57], which contains simulated data produced using a model-based simulation program. The dataset is widely used as a baseline for comparing PdM models and estimating the Remaining Useful Life (RUL). In this case, the asset to which the PdM is applied is a simulated engine composed of a multivariate sensor stream. The NASA C-MAPSS contains four sub-datasets. The data has a tabular format. Each row is a snapshot taken during a single operational cycle, and each column represents a different variable. The first column records the engine ID, the second column records the time cycle index, the third and fifth columns are the operational settings parameters, and from the 6th to 26th columns are the different sensor reading values.

**Figure 8:**
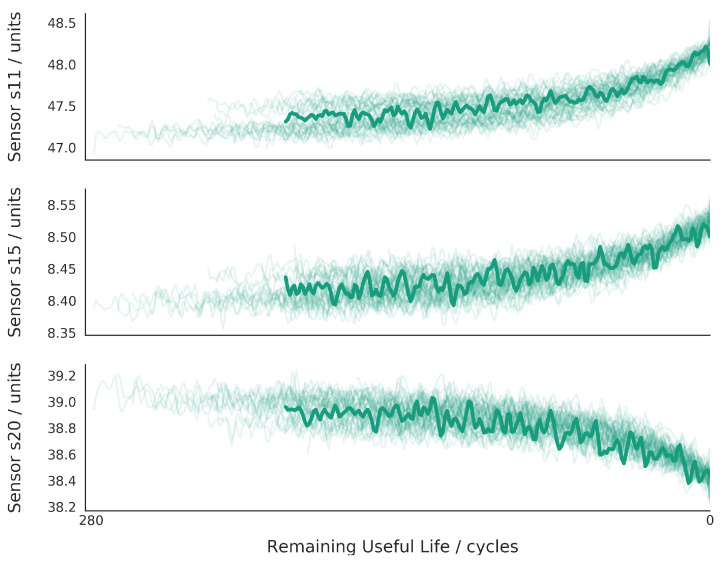
Readings of sensors 11, 15, and 20 during the engine complete lifetime training data of FD001. In the x-axis is denoted the RUL. For each sensor, several cycles of the dataset are overlaid. An example cycle is depicted in solid green.

All the engines operate at the normal condition at the start and then start to degrade during the length of the time series. An example composed of three sensor readings is depicted in Figure 8. As the number of cycles increases, the values start to deviate from the normative values represented in the first cycle iterations. Eventually, the equipment has a complete failure at RUL=0. The dataset is divided into training and test subsets. The degradation in the training set occurs until failure. Degradation at the test set ends before complete failure. Thus, the objective is to estimate the RUL.

### 4.2. Preprocessing

Before providing data to the models, we computed the pairwise Pearson correlation coefficient, ρ, to identify correlated sensor columns for each sub-dataset. We considered that two sensor streams were correlated if ρ≥0.95. All columns with zero variance were also removed.

Data preparation also involved structuring the data for the selected learning architecture. It was rearranged into a tensor with dimensions [samples, time window, features], where samples was defined as the number of sensor measurements performed; time window, τ, as the number of previous observations used for each prediction; and features the number of sensors and settings. According to the experiment performed by [30] on the effect of tau on the network performance, larger window sizes result in better RUL estimations. The authors suggested a value of τ=30 as an adequate tradeoff value between error reduction and the computational load. We followed these findings and set τ=30 in our experiment. Finally, the values were normalized to the interval [−1,1].

### 4.3. Model

The Edge TPU only supports models that are fully 8-bit quantized and then compiled specifically for the Edge TPU. Although this limitation might not significantly impact computer vision tasks, which has been the core application scenario of the Coral device, some considerations were taken into account to handle time series data, which were represented in the float32 format. Therefore, the following design considerations were considered.

#### 4.3.1. Quantization

Models can be made even smaller and more efficient through quantization, which converts 32-bit parameter data into 8-bit representations according to the Edge TPU requirements.

Two distinct strategies were employed to ensure that the inference can be performed exclusively on the Edge TPU to allow for greater execution speeds.

- **Post-training quantization** is applied after a complete model with float32 weights and activations is trained. The technique requires a representative dataset to be passed to allow the quantization process to measure the dynamic range of activations and inputs, which is critical to finding an accurate 8-bit representation of each weight and activation value. The process provides a scale and bias value, which are used to re-scale data to the int8 format [58]. After that, the model is compiled to the suitable format using the provided edgetpu_compiler to be read by the Edge TPU. The input values have to be re-scaled according to the following (Equation (1)) affine mapping of real numbers *r* to integers *q*:
  (1) $$q=\frac{r}{S}+Z$$
  where the constants *S* and *Z*, correspond to the scale and bias, respectively.
- **Quantization-aware training** requires training a model that emulates the inference quantization loss during training. The inference process is identical to the post-training quantization and requires the input data to be converted to the int8 format.

Alternatively, it is possible to perform hybrid inference, which involves executing operations that are not compatible with the Edge TPU on the Central Processing Unit (CPU). However, apart from offering potentially slower execution times, this may also lead to overhead due to the communication between the two parts.

#### 4.3.2. Architecture

The Edge TPU supports a specific set of neural network operations and architectures. It does not support Recurent Neural Network (RNN) or any variation thereof. Therefore, the proposed model is based on a deep CNN architecture, which is fully supported on the Edge TPU.

**Figure 9:**
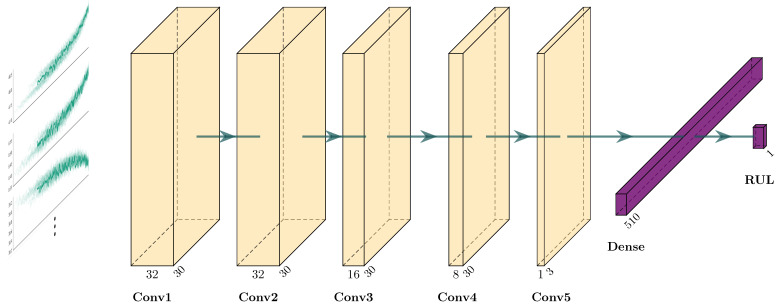
The proposed architecture for RUL estimation.

Figure 9 summarizes the model architecture, which was used in post-training quantization and quantization-aware training. The developed model is comprised of four 2-dimensional CNN layers with 32, 32, 16, and 8 filters. In the case of each filter, the kernel size is set to [30, 1]. Although 2-dimensional CNN layers are used, the convolution operation is effectively performed in one dimension, corresponding to the time-sequence. This is due to the exclusively 2-dimensional implementation of CNN layers on the Edge TPU. Another TPU layer with one filter and a kernel size of 3 × 1 is used to combine previous feature maps into one. To ensure comparable results between the two quantization strategies, all layers use the linear activation function.

In the case of the quantization-aware model, the activation function is set to linear, since tanh is not implemented by tfmot (https://www.tensorflow.org/model_optimization/api_docs/python/tfmot, accessed on 30 June 2021), the library responsible for the quantization of the model. The dropout was set to 0.2 to reduce overfitting. The resulting feature map is flattened out and combined into a single output neuron through linear activation.

The Xavier normal initializer was employed for the weight initializations [59]. For training, we used the Mean Squared Error (MSE) as the loss function and Adam [60] as the optimizer. The model was trained over 200 epochs with a batch size of 200. An early stopping strategy following the MSE loss of a validation dataset, comprising 10% of the training set, was used to prevent model overfitting.

### 4.4. Results

The results comprise the model performance, computational time, and memory consumption of the approaches with (and without) the Edge TPU. This section also discusses the results with comparisons to related state-of-the-art results on the C-MAPSS dataset. The experimental results were averaged from 30 trials to reduce the effect of randomness, and the mean values and standard deviations are provided.

The experimental results are shown in Table 1. We compared the proposed model architecture in several scenarios: the uncompressed models on the GPU (NVIDIA GeForce GTX 1650) and CPU (Intel(R) Core(TM) i7-9750H CPU @ 2.60 GHz), the compressed model using TensorFlow Lite on CPU and the compressed model on TPU with the two quantization strategies. All experiments were performed using online inference.

The most common metric for comparison for similar RUL regression models in the literature is the RMSE. Our model outperformed several CNN implementations, such as Sateesh Babu et al. [61], in all sub-datasets and offered similar performance to [30], especially in the sub-datasets RUL002 and RUL004.

Other architectures for RUL estimation include RNNs with LSTM layers [62,63], Deep Belief Networks [64], and semi-supervised deep learning [65]. Although RNNs are commonly applied to time series problems, our CNN model is particularly competitive on the sub-datasets RUL002 and RUL004, where it outperformed Zheng et al. [62].

More recent models, such as the ones presented in Listou Ellefsen et al. [65] and Al-Dulaimi et al. [33], leverage more complex hybrid approaches and offer results with lower RMSE values. However these architectures still cannot be implemented on the Edge TPU framework. Therefore, achieving better results in terms of the inference error proved to be difficult given the limitations of the aforementioned device.

Furthermore, the main objective of our research was the reduction of the inference time to enable inference on an edge computing system. Although the post-training quantization procedure may lead to larger errors compared to the uncompressed model, both models developed for the Edge TPU dataset provided similar performance on all four sub-datasets, while being approximately 4× and 2× faster than the uncompressed model inference on the CPU and GPU, respectively.

The quantization-aware model offered only slightly worse RMSE results on all sub-datasets, except for RUL003, where its performance was superior. Overall, the performance of both quantization strategies was similar. The post-quantization strategy performed slightly better on RUL001, RUL002, and RUL004, with the quantization-aware offering better results on RUL003. The inference execution times were similar.

Table 2 summarizes the file size from the resulting models using the post-quantization strategy. Values for the quantization-aware were similar and are, therefore, omitted. Both the compressed and quantized TensorFlow Lite models represent a significant reduction in comparison to the full ones. The models compiled for the Edge TPU are significantly larger in size, especially in the case of RUL003. Similar behaviour has been reported by several users and is under investigation by the development team. Nevertheless, the on-chip RAM memory (8 MB) was sufficient to accommodate the full model and keep the inference time low.

To conclude the results analysis, Table 3 presents the RMSE, MAPE, and inference time of the defined evaluation setup using the TIP4.0 default hardware—ClearFog Base (Marvell ARMADA CPU based on a 1.6 GHz A388 Dual core Arm Cortex A9)—instead of the laptop. The evaluation setup followed the procedures detailed above, with the difference that, in the ClearFog CPU, the algorithm, in some situations, entered in an overflow state that produced erroneous values. We discarded the results from these runs, since they were easily identified, presenting standard deviations with magnitudes between the hundreds and thousands.

An initial analysis of this situation indicates that the issue might be related with the different CPU architectures. The laptop used a 64 bit CPU, while the ClearFog was a 32 bit CPU. The algorithm was initially developed and tested in a 64-bit setup; therefore, running it in a different architecture might lead to overflow in certain operations. This issue is currently under investigation to guarantee an overflow-free implementation of the algorithm compatible with both CPU architectures.

Excluding these outliers, the results show that the introduction of the TPU considerably improved the inference time. In RUL001, both TPU approaches executed the inference for 9% of the time, while in RUL003 for 10% of the time, and in RUL002 and RUL004 for 15% of the time. The RMSE and MAPE presented slight variations between the CPU and TPU approaches, with no clear indication regarding which one was the most precise. The approach with the lowest RMSE and MAPE varied with the dataset. Considering this, we conclude that the introduction of an inference computing element in the edge is an added value with improvements in the processing time, and thus indirectly in the power consumption, with a negligible impact on the algorithm performance when running on the edge CPU.

Compared with the results of Table 1, there was a performance degradation in terms of the RMSE and inference time. The degradation on the RMSE might be due to the different CPU architectures (X86 64 bits vs. ARM 32 bits) and the optimization that the tensor flow run-time had for each of them. Such differences might influence the way the model is executed on each architecture, which affects the RMSE.

Regarding the inference time, the degradation might be due to ClearFog’s CPU, which was slower than the one of the laptop, and therefore all the operation and communication performed by it would be an overhead to the inference time. Additionally, since, in the ClearFog, we are running the model integrated with TIP4.0, the TPU is blank when the system starts, and thus the model needs to be uploaded to the TPU, which is also an overhead.

Regarding the MAPE, the results in Table 3 present results that are slightly better than in Table 1. This is related to the RMSE and MAPE calculations. The RMSE is calculated with the square of the error (the difference between the “real” value and the predicted value); therefore, RMSE “amplifies” the error. MAPE does not perform the square of the error and, thus, does not amplify it. Following this rationale, the results suggest that, from the set of 30 runs, some of them had higher errors, which the RMSE amplified, and in turn, resulted in an RMSE worse than the MAPE.

Considering this, the usage of the TPU for edge computing is an interesting approach to have efficient operations at the edge. However, an analysis of edge vs. cloud/server should be performed to assess if the inference performance degradation is negligible considering the improvements that edge computing can bring to the system as a whole (e.g., reduced bandwidth and memory usage and possible faster response time by having the data being processed close to its source). Further studies on edge inference performance degradation can also be performed to understand if it can be minimized.

In conclusion, TIP4.0 software architecture, its support for Google edge TPU, and the capability to perform time series analysis on the edge TPU, are an evidence of TIP4.0 support for edge computing installation and predictive maintenance scenarios.

## 5. Conclusions

We have introduced TIP4.0, which is based on COTS hardware and a modular software architecture managed by the Yocto system allowing it to be applied in predictive maintenance scenarios with and without edge computing requirements.

TIP4.0 allows for two different products for these application scenarios that are based in a single solution where each software and hardware component is selectively activated for each deployment. An AI accelerator was integrated in the solution—precisely, the Google Coral USB Accelerator. This improved the single board computer processing capabilities, which, allied with the TIP4.0 software architecture, allowed addressing edge computing deployment scenarios. This configuration was validated in laboratory conditions using the NASA C-MAPSS dataset [57].

The prior work using the Coral TPU focused on computer vision tasks. In this work, we introduced a feasibility study to process time series data. Despite some of the limitations regarding the supported operations, our proposed CNN architecture had competitive performance in comparison to the state-of-the-art alternatives, with faster execution times. This is particularly important for scenarios with large datasets and low-latency requirements, as the TPU offers the means to distributed large-scale Edge computing over industrial scenarios. The feasibility study tested the obtained algorithm in multiple settings, namely: GPU, Laptop CPU, Laptop CPU plus TPU, ClearFog CPU, and ClearFog CPU plus TPU. The TPU setups were tested with post quantization and quantization-aware models.

An overview on the how to prepare a predictive maintenance solution from the deployment point of view was also addressed in this work. This information was missing in the scientific literature and will be useful for the planning and implementation of prospective PdM solutions.

## Figures and Tables

**Table 1:** Experimental results from different model–device combinations. The evaluation metrics were the RMSE, MAPE, and the inference time reported as the mean ± standard deviation across 30 repetitions. The best results are in bold.

Dataset	Model	RMSE (Cycles)	MAPE (%)	Inference Time (s)
	Type of Model (Device)	μ ± σ	μ ± σ	μ ± σ
	Full Model (GPU)	17.03 ± 0.36	18.99 ± 0.55	19.21 ± 0.33
	Full Model (CPU)	17.03 ± 0.36	18.99 ± 0.55	32.41 ± 1.84
**RUL001**	tflite (CPU)	17.03 ± 0.36	18.99 ± 0.55	21.19 ± 0.65
	tflite-Post Quantization (Edge TPU)	17.03 ± 0.25	19.09 ± 0.48	7.23 ± 0.43
	tflite-Quantization Aware (Edge TPU)	**16.80 ± 1.16**	**18.13 ± 1.47**	**6.94 ± 0.38**
	Full Model (GPU)	**23.63 ± 0.68**	29.04 ± 0.59	36.63 ± 0.70
	Full Model (CPU)	**23.63 ± 0.68**	29.04 ± 0.59	63.46 ± 3.25
**RUL002**	tflite (CPU)	**23.63 ± 0.68**	29.04 ± 0.59	24.15 ± 1.42
	tflite-Post Quantization (Edge TPU)	23.80 ± 0.81	**29.03 ± 0.55**	17.90 ± 0.77
	tflite-Quantization Aware (Edge TPU)	24.42 ± 1.69	29.85 ± 1.83	**17.60 ± 0.68**
	Full Model (GPU)	15.55 ± 0.41	15.08 ± 0.65	19.85 ± 0.39
	Full Model (CPU)	15.55 ± 0.41	15.08 ± 0.65	46.96 ± 2.63
**RUL003**	tflite (CPU)	15.55 ± 0.41	15.08 ± 0.65	26.91 ± 1.28
	tflite-Post Quantization (Edge TPU)	15.56 ± 0.37	15.15 ± 0.60	10.51 ± 0.46
	tflite-Quantization Aware (Edge TPU)	**14.88 ± 0.91**	**14.21 ± 1.16**	**10.21 ± 0.60**
	Full Model (GPU)	**23.34 ± 0.63**	**26.18 ± 0.63**	48.84 ± 1.96
	Full Model (CPU)	**23.34 ± 0.63**	**26.18 ± 0.63**	75.78 ± 3.74
**RUL004**	tflite (CPU)	**23.34 ± 0.63**	**26.18 ± 0.63**	26.45 ± 1.83
	tflite-Post Quantization (Edge TPU)	23.37 ± 0.65	26.21 ± 0.65	22.60 ± 0.99
	tflite-Quantization Aware (Edge TPU)	23.63 ± 0.96	26.27 ± 0.86	**22.16 ± 0.86**

**Table 2:** File sizes of the full TensorFlow, TensorflowLite, and quantized TensorflowLite models in kB.

	RUL001	RUL002	RUL003	RUL004
TensorFlow (full model)	662	659	662	659
Tensorflow Lite	205	204	205	204
Quantized Tensorflow Lite	58	58	58	58
Edge TPU	121	469	1045	469

**Table 3:** Experimental results using the TIP4.0 default hardware (ClearFog Lite). The evaluation metrics were the RMSE, MAPE, and the inference time reported as the mean ± standard deviation across 30 repetitions. The best results are in bold.

Dataset	Model	RMSE (Cycles)	MAPE (%)	Inference Time (s)
	Type of Model (Device)	μ ± σ	μ ± σ	μ ± σ
**RUL001**	tflite (CPU)	19.18 ± 0.76	17.22 ± 0.68	183.10 ± 1.43
	tflite - Post Quantization (Edge TPU)	19.32 ± 0.74	17.26 ± 0.64	**16.70 ± 0.06**
	tflite - Quantization Aware (Edge TPU)	**18.13 ± 1.45**	**16.80 ± 1.14**	16.98 ± 0.08
**RUL002**	tflite (CPU)	29.04 ± 0.57	**23.63 ± 0.67**	293.61 ± 0.54
	tflite - Post Quantization (Edge TPU)	**29.03 ± 0.55**	23.80 ± 0.81	**44.60 ± 0.22**
	tflite - Quantization Aware (Edge TPU)	30.58 ± 4.16	25.39 ± 4.44	45.29 ± 0.18
**RUL003**	tflite (CPU)	15.30 ± 1.06	15.76 ± 0.89	226.26 ± 0.32
	tflite - Post Quantization (Edge TPU)	**12.05 ± 0.48**	**13.20 ± 0.42**	23.68 ± 0.11
	tflite - Quantization Aware (Edge TPU)	14.39 ± 1.33	15.04 ± 1.06	**23.67 ± 0.10**
**RUL004**	tflite (CPU)	**26.18 ± 0.62**	**23.34 ± 0.62**	368.13 ± 0.58
	tflite - Post Quantization (Edge TPU)	26.21 ± 0.65	23.37 ± 0.65	56.79 ± 0.22
	tflite - Quantization Aware (Edge TPU)	26.38 ± 1.02	23.81 ± 1.31	**57.58 ± 0.26**

## Data Availability

Not applicable.
